# Prevalence and associations of sarcopenia, obesity and sarcopenic obesity in end-stage knee osteoarthritis patients

**DOI:** 10.1186/s41043-023-00438-7

**Published:** 2023-10-13

**Authors:** Junyi Liao, Jie Chen, Wei Xu, Jia Chen, Xi Liang, Qiang Cheng, Yongli Tang, Wei Huang

**Affiliations:** 1 Department of Orthopedic Surgery, The First Affiliated Hospital of Chongqing Medical University, Chongqing, 400016 China; 2 Orthopedic Laboratory of Chongqing Medical University, Chongqing, 400016 China; 3 Department of Nutriology, The First Affiliated Hospital of Chongqing Medical University, Chongqing, 400016 China

**Keywords:** Osteoarthritis, Sarcopenia, Obesity, Sarcopenic obesity, Body composition

## Abstract

**Objective:**

To identify the prevalence of obesity, sarcopenia, sarcopenic obesity in end-stage knee osteoarthritis (KOA) patients and analyze influences of obesity and sarcopenia in the progression of KOA.

**Methods:**

A cross-sectional study was carried out among end-stage KOA patients who consecutively admitted to Orthopedic Department for TKA. We suppose that the level of decreased physical activities would be influenced by unilateral or bilateral KOA. Patient information, albumin, hemoglobin, pace, step frequency, number of comorbid conditions were collected. Bioelectrical impedance analyzer was used to analyze body composition. Obesity, sarcopenia, sarcopenic obesity rate were analyzed with accepted diagnosis criteria. Correlations between body mass index (BMI) or age and fat mass (FM), appendicular skeletal muscle mass (ASM) were analyzed.

**Results:**

138 patients (male 30, female 108) in southwest of China including 67 patients with unilateral KOA and 71 patients with bilateral KOA were analyzed. No statistic difference was found in mean albumin, prealbumin and hematocrystallin, body composition values and number of comorbid conditions. We found that BMI was positively correlated with FM (Male: R^2^ = 0.7177, *p* < 0.0001, Female: R^2^ = 0.8898, *p* < 0.0001), ASM (Male: R^2^ = 0.2640, *p* = 0.0037, Female: R^2^ = 0.2102, *p* < 0.0001), FM index (FMI) (Male: R^2^ = 0.6778, *p* < 0.0001, Female: R^2^ = 0.8801, *p* < 0.0001), and ASM index (ASMI) (Male: R^2^ = 0.3600, *p* = 0.0005, Female: R^2^ = 0.4208, *p* < 0.0001) in end-stage KOA patients. However, age was not obviously correlated with FM or FMI (Male: FM, R^2^ = 0.006911, *p* = 0.3924; FMI, R^2^ = 0.7554, *p* = 0.0009196; Female: FM, R^2^ = 0.001548, *p* = 0.8412; FMI, R^2^ = 0.002776, *p* = 0.7822). And slightly negatively correlated with ASM (Male: R^2^ = 0.05613, *p* = 0.0136, Female: R^2^ = 0.01327, *p* = 0.5433) and ASMI (Male: R^2^ = 0.02982, *p* = 0.3615; Female: R^2^ = 0.03696, *p* = 0.0462). The prevalence of obesity, sarcopenia and obesity sarcopenia differs according to different diagnosis criteria. No difference in the occurrence rate of obesity was found between bilateral KOA and unilateral KOA patients, and occurrence rates of sarcopenia and sarcopenic obesity were statistically higher in bilateral KOA than that in unilateral KOA patients.

**Conclusions:**

Obesity, sarcopenia and sarcopenic obesity are highly prevalent in end-stage KOA patients, sarcopenic obesity are more prevalent in bilateral KOA patients than that in unilateral KOA patients.

## Introduction

Osteoarthritis (OA) is a degenerative joint disease, which is characterized by wear and tear and progressive loss of articular cartilage. Late-stage OA leads to disable condition that represents a substantial and increasing health burden with notable implications for individual affected, health-care systems, and wider socioeconomic costs [1, 2]. Worldwide, an estimated more than 240 million persons have symptomatic, activity-limiting OA, in which knee osteoarthritis (KOA) contribute the most of the overall burden [3, 4]. With the aging of population, increasing of obesity and joint injuries, symptomatic KOA increases gradually [1, 3, 4]. Current pharmacologic treatment such as non-steroidal anti-inflammatory drugs (NSIADs) mainly focus on pain relief, however, disease-modifying treatment is not yet available. As for late-stage KOA, total knee arthroplasty (TKA) is the main treatment method. It is known that the key treatments of KOA are education, exercise, and weight loss if needed [1, 4, 5].

As a degenerative disease of old age, many risk factors are associated with the prevalence of KOA, which including genetic factors, age, gender, immunometabolism, obesity, dyslipidaemia, hyperglycaemia and insulin resistance and dietary factors [6–8]. Obesity and sarcopenia are the two main syndromes caused by these risk factors, which are highly prevalent in the elderly population [9, 10]. Obesity is characterized by abnormal or excessive fat accumulation, and sarcopenia is characterized by loss of muscle mass and function, the relationship between these two syndromes is not clear. Recently, sarcopenic obesity, a phenotype of low muscle mass and high adiposity was identified as a bridge between obesity and sarcopenia, which may apply new guideline for the prevention of KOA [11–13].

Obesity is positively correlated with the development of KOA. Compared with normal weight individuals, obese individuals have 2.5–4.5 times increased risk for developing KOA. Meanwhile, overweight individuals are 1.5–2.5 times more likely to develop KOA than normal weight individuals [14–16]. On the other hand, most end-stage KOA patients are aged people with dramatically decreased physical activities, which resulted in the prevalence of sarcopenia in this population [3, 11, 13, 17–19]. We suppose that the level of decreased physical activities would be influenced by unilateral or bilateral KOA, which would result in different phenotypes among sarcopenia, obesity and sarcopenic obesity in KOA cohort. On the other hand, different diagnostic criterion may lead to different portion among these three types [9, 20]. The current study analyzed the prevalence of obesity, sarcopenia and sarcopenic obesity in end-stage KOA patients, and analyzed with different diagnostic criterion, through comparing nutrition associated indicators in unilateral and bilateral KOA patients, we found that sarcopenic obesity seems more prevalent in bilateral KOA patients.

## Materials and methods

This is a cross-sectional study on end-stage KOA patients who consecutively admitted to Orthopedic Department of The First Affiliated Hospital of Chongqing Medical University for TKA. One hundred and forty-five (male 32, female 113) patients were participated this study. Patients diagnosed as rheumatoid arthritis (RA), traumatic arthritis, malignant tumor or other disease or condition which may affect the body fluid balance were excluded from the study. Finally, 138 patients (male 30, female 108) including 67 patients with unilateral KOA and 71 patients with bilateral KOA were analyzed. This study was approved by The Ethics Committee of The First Affiliated Hospital of Chongqing Medical University (NO. 2019-015) and informed consent was obtained from all the participants.

Patient information, including age, gender, weight, height and comorbid conditions were obtained from electronic medical record system. Prealbumin, albumin and hemoglobin were evaluated before surgery. Body composition was detected by Direct Segmental Multi-Frequency Bioelectrical Impedance Analyzer (DSM-BIA, Inbody 720, Korea) before surgery. Two hours before the determination, participants neither consume any liquids or solids nor do any intense activities. The same experienced technician performed the measurement for all participants as follows: supine position with bilateral ankle and wrist exposed, electrodes were placed on hairless sites of both left and right hands and feet. Phase angle, resistance, reactance, intracellular water (ICW), extracellular water (ECW), total body water (TBW), soft lean mass (SLM), fat free mass (FFM), skeletal muscle mass (SMM), body cell mass (BCM), Mineral, Bone Mineral Content, Waist Circle (Waist Cir.), basal metabolic rate (BMR), fat mass (FM), percent body fat (PBF) and visceral fat area (VFA), were measured in different frequency, as described previously [10, 21–23]. Step frequency and pace were calculated by recording time and frequency when patients walk 20 m.

Statistical analysis was performed with GraphPad Prism (GraphPad Software, La Jolla, CA) version 9.0. software. Quantitative data are shown as mean ± standard deviation (SD). There were no missing data on the patients included in the analyses. Normality of continuous variable was tested by Shapiroe-Wilk test. Between-group comparisons were conducted using Student's independent t-test, Chi-square, or Fisher's exact test, as appropriate, based on the distribution, variable type, and number in each group. Correlations were analyzed with simple linear regression. A two-tailed *p* value < 0.05 were considered statistically significant.

## Results

**Fig. 1:**
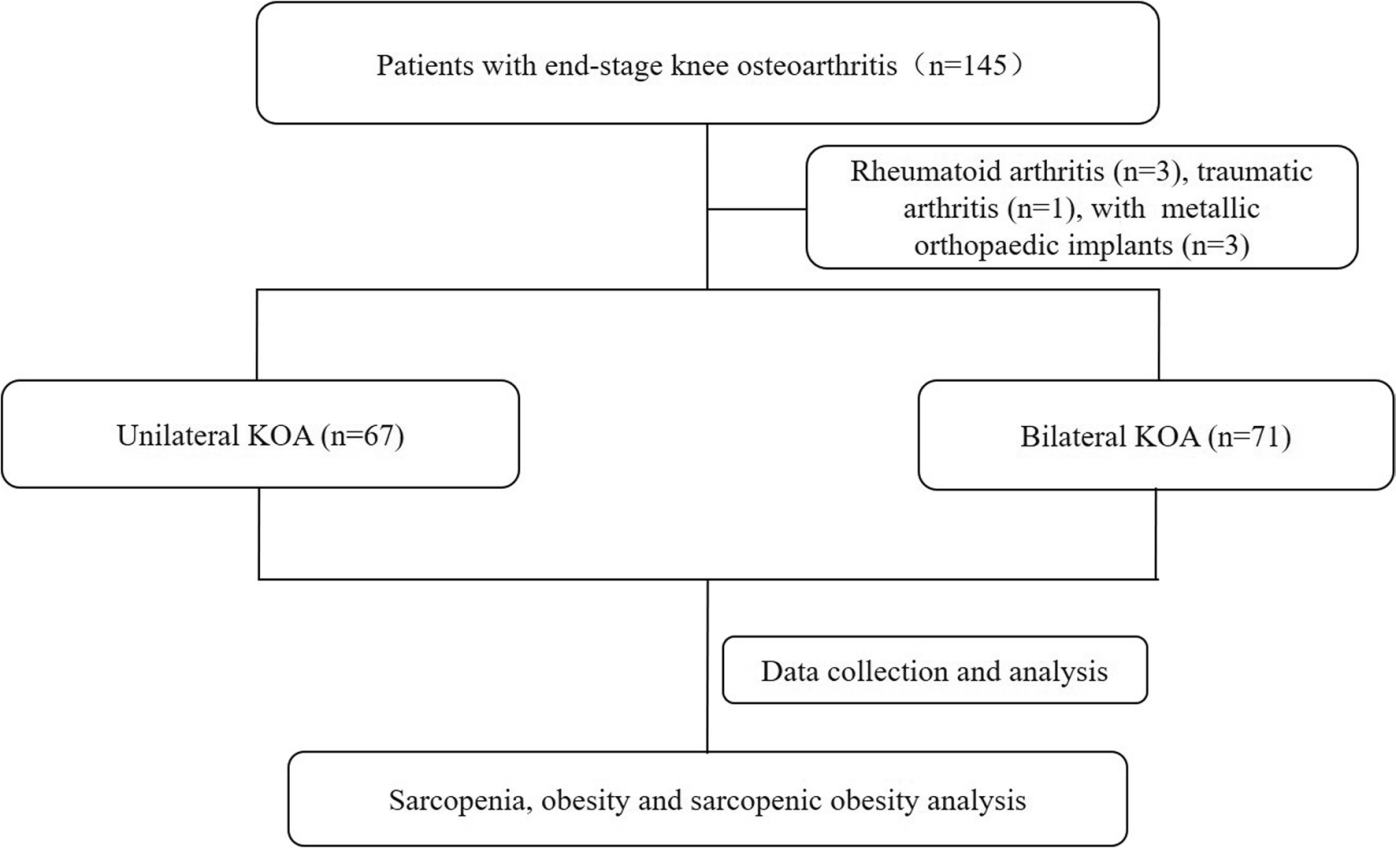
Flow chart of subject selection. KOA: knee osteoarthritis

 Flow chart of the current study was shown in Fig. 1, 138 patients diagnosed as end-stage KOA were included for the analysis, including 67 patients with unilateral KOA and 71 patients with bilateral KOA. Unilateral KOA group including 54 females and 13 males, and bilateral KOA group including 54 females and 17 males. Step frequency and pace in unilateral KOA were statistic higher than bilateral KOA patients (*p* < 0.0001). Patient characteristics were listed in Table 1, there were no statistic difference in mean age and BMI. No statistic difference was found in mean albumin, prealbumin and hematocrystallin values. Number of comorbid conditions and types of comorbid conditions were also listed in Table 1.

**Table 1:** Patients characteristics of unilateral and bilateral knee osteoarthritis (KOA)

	Unilateral KOA		Bilateral KOA		*p*
	Mean ± SD	n	Mean ± SD	n	
Age (year)	68.70 ± 9.62	67	68.07 ± 6.88	71	*0.6575*
Body mass index (kg/m^2^)	25.78 ± 3.69	67	25.19 ± 3.68	71	*0.3489*
Step frequency	79.14 ± 14.40	67	68.45 ± 7.52	71	< 0.0001****
Pace	1.02 ± 0.22	67	0.75 ± 0.095	71	< 0.0001****
Gender	*/*		/		*/*
Female	*/*	54	/	54	*/*
Male	*/*	13	/	17	*/*
Albumin (g/L)	41.00 ± 4.82	67	40.56 ± 3.60	71	*0.5430*
Prealbumin (g/L)	221.55 ± 52.56	67	226.70 ± 44.52	71	*0.5348*
Hematocrystallin (g/L)	122.24 ± 16.35	67	124.92 ± 14.61	71	*0.3112*
Number of comorbid conditions	/	54/67	/	55/71	/
Types of comorbid conditions					
Type II diabetes	/	12	/	7	/
Dyslipidemia	/	16	/	22	/
Hypertension	/	20	/	17	/
Cardiovascular disease	/	4	/	4	/
Other chronic diseases	/	36	/	34	/

Italic values indicates *p* values, the values with *indicates statistic significance, and *indicates *p* < 0.05, **indicates *p* < 0.01,***indicates *p* < 0.001, ****indicates *p* < 0.0001

BMI screens for weight categories that may lead to healthy problems. We firstly analyzed BMI distribution according to underweight (less than 18.0 kg/m^2^), normal weight (18–24.99 kg/m^2^), overweight (25.0–30.0 kg/m^2^) and obese (more than 30 kg/m^2^). As presented in Table 2, more than half (42 in 67 in unilateral and 39 in 71 in bilateral KOA) participates were classified to overweight or obesity, although no statistic difference was found when compare unilateral KOA group and bilateral KOA group according to BMI classification respectively. These results indicated the prevalence of obesity in KOA patients.

**Table 2:** BMI classification of unilateral and bilateral KOA patients

BMI	Unilateral KOA	Bilateral KOA	*X*^2^	*p*
< 18	0% (0/67)	2.82% (2/71)	1.915	*0.1664*
18–24.99 kg/m^2^	37.31% (25/67)	42.25% (30/71)	0.3509	*0.5536*
25.0–30.0 kg/m^2^	52.24% (35/67)	47.89% (34/71)	0.2611	*0.6904*
> 30 kg/m^2^	10.45% (7/67)	7.04% (5/71)	0.5035	*0.4780*

Italic values indicates *p* values, the values with *indicates statistic significance, and *indicates *p* < 0.05, **indicates *p* < 0.01,***indicates *p* < 0.001, ****indicates *p* < 0.0001

To further evaluate the nutritional condition of participates, body composition was detected by BIA. There was no significant statistic difference between unilateral KOA group and bilateral KOA group in BMR, ICW, ECW, TBW, SLM, FFM, SMM, ECW/TBW, BCM, protein, mineral, bone mineral content, Waist cir., Arm Cir., and Arm muscle Cir. (Table 3), which suggested no dramatic difference was exist for single nutritional marker.

**Table 3:** Basal Metabolic Rate (BMR) and body composition parameters in Unilateral and bilateral KOA patients

	Unilateral KOA	Bilateral KOA	*t*	*p*
	Mean ± SD	Mean ± SD		
BMR (Basal metabolic rate) (kcal)	1192.61 ± 151.70	1245.74 ± 160.08	*1.999*	*0.04763*
ICW (Intracellular water) (L)	18.44 ± 32.88	17.58 ± 2.77	*0.2196*	*0.8265*
ECW (Extracellular water) (L)	11.85 ± 1.84	11.39 ± 1.67	*1.539*	*0.1261*
TBW (Total Body water) (L)	30.29 ± 4.70	28.98 ± 4.43	*1.686*	*0.09417*
SLM (Soft lean mass) (kg)	38.75 ± 6.01	37.03 ± 5.69	*1.727*	*0.08644*
FFM (Fat free mass) (kg)	41.09 ± 6.33	39.28 ± 5.95	*1.732*	*0.08562*
SMM (Skeletal muscle mass) (kg)	22.04 ± 3.75	20.94 ± 3.62	*1.753*	*0.08181*
ECW/TBW	0.39 ± 0.0090	0.39 ± 0.0084	*0*	> *0.9999*
BCM (Body cell mass) (kg)	26.41 ± 4.12	25.19 ± 3.97	*1.771*	*0.07872*
Protein (kg)	7.98 ± 1.24	7.61 ± 1.21	*1.774*	*0.07833*
Mineral (kg)	2.83 ± 0.41	2.71 ± 0.34	*1.876*	*0.06283*
Bone mineral content (kg)	2.34 ± 0.39	2.24 ± 0.28	*1.738*	*0.08454*
Waist cir. (cm)	86.81 ± 9.86	85.35 ± 9.16	*0.9017*	*0.3688*
Arm cir. (cm)	30.75 ± 2.97	30.34 ± 2.87	*0.8247*	*0.4110*
Arm muscle cir. (cm)	23.86 ± 1.88	23.53 ± 1.81	*1.051*	*0.2953*
Fat (kg)	23.05 ± 7.44	22.58 ± 7.32	0.374	*0.7090*
Visceral fat area (VFA) (cm^2^)	119.19 ± 43.16	122.15 ± 46.84	0.3854	*0.7005*

Italic values indicates *p* values, the values with *indicates statistic significance, and *indicates *p* < 0.05, **indicates *p* < 0.01,***indicates *p* < 0.001, ****indicates *p* < 0.0001

**Fig. 2:**
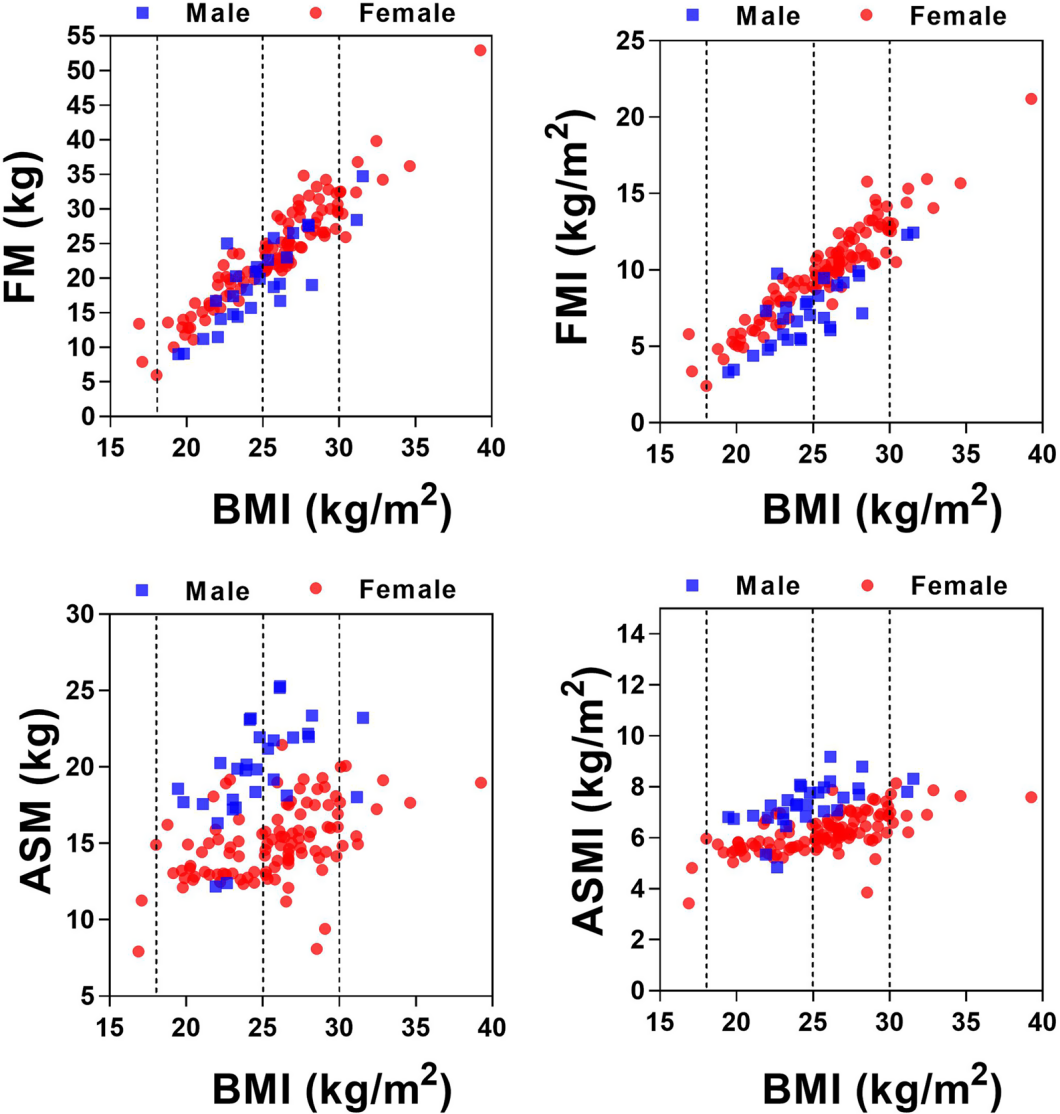
Correlation between FM, FMI, ASM, ASMI and BMI. FM: fat mass, FMI: fat mass index, ASM: appendicular skeletal muscle, ASMI: appendicular skeletal muscle mass index

In different stage of KOA, body composition would change accordingly. Therefore, based on the cross-sectional data, we analyzed relationships between BMI and fat mass (FM), fat mass index (FMI), appendicular skeletal muscle mass (ASM) and appendicular skeletal muscle mass index (ASMI) respectively. As shown in Fig. 2, with the increasing of BMI, FM (Male: R^2^ = 0.7177, *p* < 0.0001, slope 95% CI 1.359–2.230, Female: R^2^ = 0.8898, *p* < 0.0001, slope 95% CI 1.676–1.933) and FMI (Male: R^2^ = 0.6778, *p* < 0.0001, slope 95% CI 0.4776–0.8255, Female: R^2^ = 0.8801, *p* < 0.0001, slope 95% CI 0.7105–0.8138) increased accordingly. Meanwhile, with the increasing of BMI, ASM (Male: R^2^ = 0.2640, *p* = 0.0037, slope 95% CI 0.2008–0.9349, Female: R^2^ = 0.2102, *p* < 0.0001, slope 95% CI 0.1828–0.4004) and ASMI (Male: R^2^ = 0.3600, *p* = 0.0005, slope 95% CI 0.08997–0.2819, Female: R^2^ = 0.4208, *p* < 0.0001, slope 95% CI 0.1017–0.1610) increased with lower correlation. These results indicated that with the increasing of BMI, fat mass, not appendicular skeletal muscle mass, was the main increasing in end-stage KOA patients, which imply the specific nutritional condition in end-stage KOA patients.

Appendicular skeletal muscle mass and fat mass are the two main markers that stand for the balance between muscle and fat in aspect of body composition. We firstly analyzed muscle mass in KOA patients. With the using of appendicular skeletal muscle mass index by different diagnostic criteria (ASM by height^2^, weight, and BMI), we found that the diagnosis of reduced muscle mass (sarcopenia) differs from diagnostic criteria, however, the incidence rate of reduced muscle mass was higher in bilateral KOA than that in unilateral KOA patients (Table 4). As for the prevalence of obesity, we also use different diagnostic criteria (by BMI, Waist Cir, and PBF), the results showed that in incidence rate of obesity differs from different diagnostic criteria, with the lowest incidence rate by BMI (10.45% in Unilateral KOA and 7.04% in Bilateral KOA) and the highest incidence rate by Waist Cir. (74.63% in Unilateral KOA and 67.61% in Bilateral KOA). However, there was no statistic difference between unilateral KOA and bilateral KOA for the incidence rate of obesity with different diagnostic criteria (Table 5).

**Table 4:** Prevalence of reduced muscle mass by diagnostic criteria

	Unilateral KOA	Bilateral KOA	*X*^*2*^	*p*
ASM by height^2#^				
Total, n (%)	20.90% (14/67)	32.39% (23/71)	*2.323*	*0.1275*
Female, n (%)	24.07% (13/54)	25.93% (14/54)	*0.04938*	*0.8241*
Male, n (%)	7.69% (1/13)	52.94% (9/17)	*6.787*	0.0092**
ASM by weight^#^				
Total, n (%)	4.48% (3/67)	4.23% (3/71)	*0.1994*	*0.6552*
Female, n (%)	3.70% (2/54)	1.85% (1/54)	*0.3429*	*0.5582*
Male, n (%)	7.69% (1/13)	11.76% (2/17)	*0.1357*	*0.7125*
ASM by BMI^#^				
Total, n (%)	11.94% (8/67)	30.99% (22/71)	*7.349*	0.0067**
Female, n (%)	9.26% (5/54)	24.07% (13/54)	*4.966*	0.0259*
Male, n (%)	23.08% (3/13)	52.94% (9/17)	*2.7348*	*0.0980*

Italic values indicates *p* values, the values with *indicates statistic significance, and *indicates *p* < 0.05, **indicates *p* < 0.01,***indicates *p* < 0.001, ****indicates *p* < 0.0001

*ASM* appendicular skeletal muscle mass, *BMI* body mass index

^#^Cut-off criteria for females, males, respectively: ASM by height^2^, kg/m^2^ (< 5.7, < 7.0), ASM by weight, % (< 19.43, < 25.72), ASM by BMI, kg/m^2^ (< 0.512, < 0.789)

**Table 5:** Prevalence of obesity by diagnostic criteria

	Unilateral KOA	Bilateral KOA	*X*^*2*^	*p*
Obesity by BMI^#^				
Total, n (%)	10.45% (7/67)	7.04% (5/71)	*0.5035*	*0.4780*
Female, n (%)	11.11% (6/54)	7.41% (4/54)	*0.4408*	*0.5067*
Male, n (%)	7.69% (1/13)	5.88% (1/17)	*0.0387*	*0.8439*
Obesity by waist cir.^#^				
Total, n (%)	74.63% (50/67)	67.61% (48/71)	*0.8256*	*0.3636*
Female, n (%)	79.63% (43/54)	75.93% (41/54)	*0.2143*	*0.6434*
Male, n (%)	53.85% (7/13)	41.18% (7/17)	*0.4751*	*0.4906*
Obesity by PBF^#^				
Total, n (%)	65.67% (44/67)	70.42% (50/71)	*0.3582*	*0.5495*
Female, n (%)	66.67% (36/54)	70.37% (38/54)	*0.1717*	*0.6786*
Male, n (%)	61.54% (8/13)	70.59% (12/17)	*0.2715*	*0.6023*

Italic values indicates *p* values, the values with *indicates statistic significance, and *indicates *p* < 0.05, **indicates *p* < 0.01,***indicates *p* < 0.001, ****indicates *p* < 0.0001

*BMI* body mass index, *Waist Cir.* waist circumference, *PBF* percent body fat

^#^Cut-off criteria for females, males, respectively: Obesity by BMI, kg/m^2^ (> 30, > 30), Obesity by Waist Cir., cm (> 80, > 85), Obesity by PBF, % (≥ 35, ≥ 25)

To further analyze the incidence of sarcopenic obesity (SO) in KOA patients. We found that the prevalence of SO in the overall cohort varied according to diagnostic criteria. As shown in Table 6, a higher prevalence of SO was identified with ASM by BMI and PBF, and with the criteria of ASM by height^2^ and BMI no SO was identified. Alternatively, a higher prevalence of SO was found in bilateral male KOA patients with the criteria of ASM by height^2^ and Waist Cir., ASM by height^2^ and PBF, ASM by BMI and Waist Cir., and ASM by BMI and PBF.

**Table 6:** Prevalence of sarcopenic obesity (SC) by diagnostic criteria

	Unilateral KOA	Bilateral KOA	*X*^*2*^	*p*
ASM by height^2^ and BMI				
Total, n (%)	0 (0/67)	0 (0/71)	*/*	*/*
Female, n (%)	0 (0/54)	0 (0/54)	*/*	*/*
Male, n (%)	0 (0/13)	0 (0/17)	/	*/*
ASM by height^2^ and waist cir				
Total, n (%)	8.96% (6/67)	14.08% (10/71)	*0.8848*	*0.3469*
Female, n (%)	11.11% (6/54)	12.96% (7/54)	*0.08745*	*0.7674*
Male, n (%)	0 (0/13)	17.65% (3/17)	*1.218*	*0.2698*
ASM by height^2^ and PBF				
Total, n (%)	8.96% (6/67)	18.31% (13/71)	*2.541*	*0.1109*
Female, n (%)	11.11% (6/54)	12.96% (7/54)	*0.08745*	*0.7674*
Male, n (%)	0 (0/13)	35.29% (6/17)	*5.735*	0.0166*
ASM by weight and BMI				
Total, n (%)	2.98% (2/67)	0 (0/71)	*2.151*	*0.1425*
Female, n (%)	1.85% (1/54)	0 (0/54)	*1.009*	*0.3151*
Male, n (%)	7.69% (1/13)	0 (0/17)	*1.353*	*0.2448*
ASM by weight and waist cir				
Total, n (%)	4.48% (3/67)	2.82% (2/71)	*0.2723*	*0.6018*
Female, n (%)	3.70% (2/54)	1.85% (1/54)	*0.3429*	*0.5582*
Male, n (%)	7.69% (1/13)	5.88% (1/17)	*0.03878*	*0.8439*
ASM by weight and PBF				
Total, n (%)	4.48% (3/67)	4.23% (3/71)	*0.005275*	*0.9421*
Female, n (%)	3.70% (2/54)	1.85% (1/54)	*0.3429*	*0.5582*
Male, n (%)	7.69% (1/13)	11.76% (2/17)	*0.1357*	*0.7125*
ASM by BMI and BMI				
Total, n (%)	4.48% (3/67)	5.63% (4/71)	*0.0957*	*0.7571*
Female, n (%)	3.70% (2/54)	5.56% (3/54)	*0.2097*	*0.6470*
Male, n (%)	7.69% (1/13)	5.88% (1/17)	*0.03878*	*0.8439*
ASM by BMI and Waist Cir				
Total, n (%)	11.94% (8/67)	21.13% (15/71)	*2.095*	*0.1478*
Female, n (%)	9.26% (5/54)	18.52% (10/54)	*1.935*	*0.1642*
Male, n (%)	23.07% (3/13)	29.41% (5/17)	*0.1512*	*0.6974*
ASM by BMI and PBF				
Total, n (%)	11.94% (8/67)	28.18% (20/71)	*5.613*	0.0178*
Female, n (%)	9.26% (5/54)	22.22% (12/54)	*3.421*	*0.0644*
Male, n (%)	17.65% (3/17)	61.54% (8/13)	*6.111*	0.0134*

Italic values indicates *p* values, the values with *indicates statistic significance, and *indicates *p* < 0.05, **indicates *p* < 0.01,***indicates *p* < 0.001, ****indicates *p* < 0.0001

*BMI* body mass index, *Waist Cir.* waist circumference, *PBF* percent body fat

Cut-off criteria for females, males, respectively: Obesity by BMI, kg/m^2^ (> 30, > 30), Obesity by Waist Cir., cm (> 80, > 85), Obesity by PBF, % (≥ 35, ≥ 25)

Cut-off criteria for females, males, respectively: ASM by height^2^, kg/m^2^ (< 5.7, < 7.0), ASM by weight, % (< 19.43, < 25.72), ASM by BMI, kg/m^2^ (< 0.512, < 0.789)

**Fig. 3:**
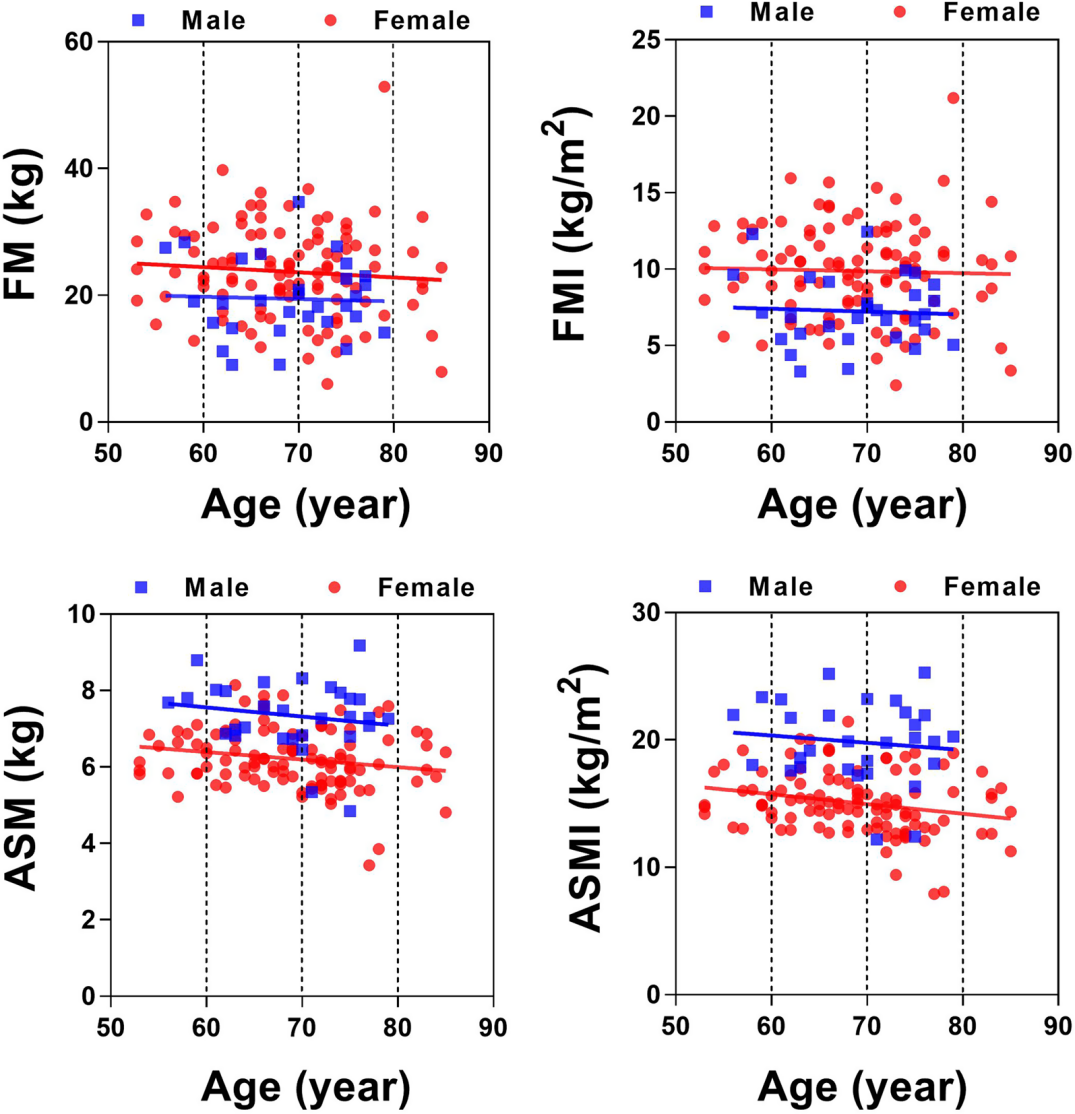
Correlation between FM, FMI, ASM, ASMI and age. FM: fat mass, FMI: fat mass index, ASM: appendicular skeletal muscle, ASMI: appendicular skeletal muscle mass index

As we previously characterized that age is positively correlated with the prevalence of sarcopenia. Here, we also analyzed correlations between ages and FM, FMI, ASM and ASMI (Fig. 3). The results showed that age was not obviously correlated with FM or FMI in both female (FM, R^2^ = 0.006911, *p* = 0.3924, slope 95% CI − 0.2683 to 0.1061; FMI, R^2^ = 0.7554, *p* = 0.0009196, slope 95% CI − 0.09130 to 0.06645) and male patients (FM, R^2^ = 0.001548, *p* = 0.8412, slope 95% CI − 0.4021 to 0.3298; FMI, R^2^ = 0.002776, *p* = 0.7822, slope 95% CI − 0.1552 to 0.1180). As for ASM and ASMI, we found that age was slightly negatively correlated with ASM and ASMI in female patients (ASM, R^2^ = 0.05613, *p* = 0.0136, slope 95% CI − 0.1368 to − 0.01607; ASMI, R^2^ = 0.03696, *p* = 0.0462, slope 95% CI − 0.03915 to − 0.0003367, and not statistically correlation was found between age and ASM (R^2^ = 0.01327, *p* = 0.5433, slope 95% CI − 0.2467 to 0.1329) and ASMI (R^2^ = 0.02982, *p* = 0.3615, slope 95% CI − 0.07668 to 0.02887) in male patients. These results suggested that age should not be the main risk factor of sarcopenia or obesity in KOA cohort.

## Discussion

With the aging of population, morbidity of KOA increases gradually. Many factors, including age, genetic factors, gender, immunometabolism and obesity are correlated with the incidence of KOA. The relationship between obesity and KOA has been commonly studied, recently the relationship between sarcopenia and end-stage KOA has been noticed and reported, some of the studies explored one phenotype named sarcopenic obesity [13]. These studies indicated the complex relationships among sarcopenia, obesity and sarcopenic obesity [6, 13, 24]. We supposed that KOA induced decreased activities may accelerate the occurrence of sarcopenic obesity and compared sarcopenic obesity between unilateral and bilateral KOA patients. Our results found there is no difference in the occurrence rate of obesity between bilateral and unilateral end-stage KOA cohort, and occurrence rates of sarcopenia and sarcopenic obesity were statistically higher in bilateral KOA than that in unilateral KOA cohort. These results indicated that decreased activity is the risk factor for sarcopenic obesity in KOA cohort, in other words, bilateral KOA cohort with a higher risk of sarcopenia obesity.

KOA is one of the most prevalent forms of knee disease and a growing cause of disability worldwide. It is reported that nutritional condition is associated with the occurrence and progression of KOA on basis of that several nutrients participated cartilage metabolism [7]. On the other hand, obesity or overweight is another risk factor of KOA and sarcopenia is prevalent in older population [6, 12, 13]. Therefore, we analyzed the prevalence of obesity and sarcopenia in end-stage KOA cohort. We found that almost half end-stage KOA patients were diagnosed as obesity or overweight according to BMI criteria, and no obvious difference was found between unilateral and bilateral KOA cohort. As for the body composition analysis, there is no statistical difference between unilateral and bilateral KOA cohort. However, bilateral KOA patients with a higher incidence of sarcopenia compared with unilateral KOA patients. These results indicated that obesity is a risk factor of KOA, and sarcopenia may arise or aggravate with KOA progression. Decreased physical activity level is one of the key features of KOA cohort, which may cause or aggravate sarcopenia [13], through analyzing bilateral and unilateral KOA patients, we found obviously decreased pace and step frequency in bilateral KOA patients compared with unilateral KOA. However, we did not find statistic association between KOA side and decreased muscle mass side, this indicates sarcopenia in KOA patients is also characterized by systematic decreasing of muscle mass, rather than KOA affected limb muscle mass. On the other hand, sarcopenia is characterized as a geriatric syndrome and is a major challenge to healthy aging, which cause worse clinical outcomes and higher mortality than those without sarcopenia [9, 25]. The high incidence rate of sarcopenia in end stage KOA cohort needs to be pay attention to during perioperative recovery and functional training, prevention and treatment of sarcopenia and obesity may be beneficial for comprehensive treatment of KOA.

It is reported that men and women present different trajectories in the decline in skeletal muscle with aging. Women tend to have a sudden drop in muscle mass following menopause and men have a gradual decline especially in in sedentary individuals [26, 27]. In this study, we found that men presented a higher incidence of sarcopenia in bilateral KOA compared with unilateral KOA patients, and no obvious difference was found in women. This phenomenon may because sarcopenia in women was associated with estrogen level and activity level, in men was mainly associated with activity level.

Age is negatively corelated with the incidence of sarcopenia [28–35]. In end-stage KOA cohort, we found that age is negatively correlated with ASM and ASMI in female patients, however with a small correlation coefficient, and no statistic correlation was found in male patients. These results indicated that in end-stage KOA cohort, other risk factors, especially decreased activities and increased BMI were the main cause of sarcopenia. Meanwhile, we characterized that overweight or obesity is another feature of end-stage KOA cohort, and increased BMI is positively correlated with the increase of FM and FMI. Therefore, be similar with the previous studies [12, 13, 18], we also identified the prevalence of sarcopenia obesity in end-stage KOA cohort. As the diagnosis of SC differs from criteria, our data showed the high incidence of obesity by Waist Cir. or PBF, rather than by BMI, which indicates the high incidence of central obesity in end-stage KOA cohort. Be different with sarcopenia, obesity rates did not differ between unilateral and bilateral cohort, which indicates that obesity is one of the primary risk factors of KOA, and sarcopenia stand for relative severe activity limitation caused by KOA. In other words, obesity is a risk factor of KOA, and KOA, especially bilateral KOA is a risk factor of sarcopenia. Since sarcopenia is associated with fragility, fracture, osteoporosis, poor surgical outcomes etc. [32, 33, 35–38], the treatment or improve of SC in end-stage KOA patients is necessary for improving functional results perioperatively.

As one of the most important non-surgical treatment methods of KOA, therapeutic exercise is strongly recommended for the treatment or prevent of KOA [39]. The main advantages of exercise including enhance muscle strength, maintain joint stability etc. [39–42]. Meanwhile, physical exercise is essential for the prevention of sarcopenia [27]. In this study, the data also indicated the prevalence of sarcopenia in KOA patients, therefore, we deduce that lack of physical exercises not only induced the prevalence of sarcopenia, but also facilitated KOA progression. On the other hand, weight loss is strongly recommended for patients with KOA who are overweight or obese [39]. We found that obesity or overweight are highly prevalent in KOA cohort, which suggested that weight loss is a potential therapeutic target for KOA prevention and treatment. Taken together, rational physical exercise is beneficial for prevention of sarcopenia, obesity, sarcopenia obesity and knee osteoarthritis.

This study has some limitations. This is a cross sectional study, and we selected all the patient in the same hospital. Our study included Chinese people only, consequently, our data are not generalizable to the overall population who sustain hip fractures. We only analyzed end-stage OA patients and compared indicators between unilateral and bilateral KOA patients. Further studies focus on improving KOA progression through decreasing the occurrence of sarcopenia and/or obesity are needed.

In conclusion, this study showed that the prevalence of obesity, sarcopenia and sarcopenic obesity in end-stage KOA patients, although results do not conclusively establish weight loss and increased muscle mass as protective factors against osteoarthritis, procedures aimed to weight loss and increase muscle mass should be beneficial for KOA prevention and treatment.

## Data Availability

All data generated in this study are included in the article or supplementary materials.
